# The Role of Combined SGLT1/SGLT2 Inhibition in Reducing the Incidence of Stroke and Myocardial Infarction in Patients with Type 2 Diabetes Mellitus

**DOI:** 10.1007/s10557-021-07291-y

**Published:** 2021-11-09

**Authors:** Bertram Pitt, Gabriel Steg, Lawrence A. Leiter, Deepak L. Bhatt

**Affiliations:** 1 University of Michigan, Ann Arbor, MI USA; 2 Université de Paris, Hopital Bichat, Paris, France; 3 Li Ka Shing Knowledge Institute, St. Michael’s Hospital, University of Toronto, Toronto, ON Canada; 4 Brigham and Women’s Hospital Heart & Vascular Center and Harvard Medical School, 75 Francis Street, Boston, MA 02115 USA

**Keywords:** Type 2 diabetes, Cardiovascular risk, Quality of life, Sodium-glucose cotransporter 1/2 inhibitors

## Abstract

**Purpose:**

In patients with type 2 diabetes mellitus (T2DM), both sodium-glucose cotransporter 2 inhibitors (SGLT2is) and glucagon-like peptide receptor agonists (GLP-1 RAs) have demonstrated significant improvements in cardiovascular and kidney outcomes independent of their glycemic benefits. This paper will briefly compare the effect of SGLT2is and GLP-1 RAs to that of the SGLT1/2 inhibitor sotagliflozin on the incidence of myocardial infarction (MI) and stroke in patients with T2DM and further postulate mechanisms to account for these findings.

**Methods and Results:**

Thus far, the results from SCORED and SOLOIST (trials studying the SGLT1/2 inhibitor sotagliflozin) suggest that an increase in SGLT1 inhibition when added to SGLT2 inhibition may contribute to reductions in MI and stroke in patients with T2DM. This benefit is beyond what SGLT2is alone can accomplish and at least similar to GLP-1 RAs but with the added benefit of a reduction in hospitalizations and urgent visits for HF. Larger and longer studies are required to confirm the effectiveness of SGLT1/SGLT2 inhibition in reducing MI and stroke in patients with T2DM and elucidate the mechanisms associated with this finding.

**Conclusions:**

The role of SGLT1/2 inhibition as an addition to GLP-1 RAs in patients with and without T2DM at increased risk for MI and stroke requires further study. Regardless, the finding that a relative increase in SGLT1/2 inhibition reduces the risk of MI and stroke as well as hospitalizations and urgent visits for heart failure could improve quality of life and reduce the healthcare burden associated with T2DM.

In patients with type 2 diabetes mellitus, both sodium-glucose cotransporter 2 inhibitors (SGLT2is) and glucagon-like peptide receptor agonists (GLP-1 RAs) have been shown to significantly improve cardiovascular and kidney outcomes independent of their effect on glycemia. Current guidelines, as well as a recent meta-analysis of SGLT2is and GLP-1 RAs, reflect the efficacy of these drug classes [1–4]. Although they significantly improve cardiovascular and kidney outcomes in patients with type 2 diabetes, their utility in preventing myocardial infarction (MI) and stroke is relatively modest and inconsistent, especially with SGLT2is.

The recent findings from the SCORED (10,584 patients with type 2 diabetes and CKD randomized to sotagliflozin or placebo) and SOLOIST (1222 patients with type 2 diabetes admitted with worsening heart failure randomized to sotagliflozin or placebo) trials suggest that like SGLT2is, SGLT1/2 s reduce the composite of deaths from cardiovascular causes, hospitalization for heart failure, and urgent visits for heart failure but may provide greater reduction in MI and stroke [5, 6]. Although there is no direct comparison of the SGLT1/2i sotagliflozin to the SGLT2is, a meta-analysis of SGLT2is has failed to show a reduction of stroke and only a modest reduction in the incidence of MI [3]. Therefore, the combination of SGLT1/SGLT2 inhibition may benefit patients with type 2 diabetes beyond what current therapeutic options can offer. Previously, we illustrated the benefit of a relative increase in SGLT1/SGLT2 inhibition with regard to reduction in stroke and MI [7]. This paper will briefly compare the effect of SGLT2is and GLP-1 RAs to that of the SGLT1/2 inhibitor sotagliflozin on the incidence of MI and stroke in patients with type 2 diabetes and further postulate mechanisms associated with SGLT1 inhibition that could account for these findings.

In general, SGLT2is reduce cardiovascular mortality, kidney outcomes, and heart failure hospitalizations (HHF). In contrast, GLP-1 RAs primarily reduce atherosclerotic events. A recent meta-analysis of 6 trials with unique individual data from 46,969 patients with type 2 diabetes found that SGLT2is significantly reduced the risk of major cardiovascular events (HR 0.90; 95% CI 0.85–0.95), HHF/cardiovascular mortality (HR 0.78; 95% CI 0.73–0.84), and kidney outcomes (HR 0.62; 95% CI 0.56–0.70) [3]. The presence or absence of atherosclerotic cardiovascular disease did not significantly modify the benefit for these adverse cardio-renal events. Overall, there was a modest reduction in MI (HR 0.91; 95% CI 0.84–0.99). In those with baseline ASCVD, MI was similarly reduced (HR 0.90; 95% CI 0.82–0.99). Thus, SGLT2is are effective in reducing the incidence of MI but their effects are modest and likely related to a reduction in myocardial oxygen demands rather than an effect on thrombosis [8]. Surprisingly, there was no overall reduction in stroke (HR 0.96; 95% CI 0.87–1.07), even in those with baseline atherosclerotic cardiovascular disease (HR 0.99; 95% CI 0.87–1.11) [3] despite the reductions in inflammatory cytokines, oxidative stress, visceral obesity, and blood pressure that occur with these agents [9]. The reasons SGLT2is fail to reduce the occurrence of MI to a greater degree and overall stroke remain uncertain, especially in view of their reduction in blood pressure which on the basis of prior epidemiologic studies should have predicted a reduction in stroke [10]. However, their resultant increase in erythropoietin and as a consequence hematocrit and blood viscosity [11], which could in part be attributed to the effect of SGLT2i to increase sodium excretion and to decrease plasma volume, could predispose to a slight increase in thrombosis [12], thereby offsetting the reduction in stroke that would otherwise be expected from the blood pressure lowering.

A meta-analysis of GLP-1 RAs from trials comprising 56,004 patients found a significant reduction in 3-point MACE (cardiovascular mortality, nonfatal MI, and nonfatal stroke) (HR 0.88, 95% CI 0.79–0.98), cardiovascular mortality (HR 0.88, 95% CI 0.76–0.94), all-cause mortality (HR 0.89, 95% CI 0.81–0.97), fatal and nonfatal stroke (HR 0.84, 95% CI 0.76–0.94), and HHF (HR 0.92, 95% CI 0.86–0.97) as well as a trend towards reduction in nonfatal and fatal MI (HR 0.91, 95% CI 0.82–1.02) [4]. In a sensitivity analysis using a less conservative statistical approach, there was significant benefit on fatal and nonfatal MI (HR 0.91, 95% CI 0.83–1.00; *p* = 0.039). The meta-analysis also found no increase in the incidence of hypoglycemia, pancreatitis, and pancreatic cancer in patients taking a GLP-1 RA compared with those on placebo [4]. However, the various GLP-1 RAs differ in structure, duration of action, and effects. The greatest reduction in fatal and nonfatal MI was seen in the HARMONY trial of albiglutide (HR 0.75; 95% CI 0.61–0.90) [13], but there was no significant reduction in fatal stroke (HR 0.86; 95% CI 0.65–1.14). SUSTAIN-6 [14] with subcutaneous semaglutide saw the greatest numerical reduction in fatal and nonfatal stroke, but it was not statistically significant (HR 0.65; 95% CI 0.41–1.03) in this smaller phase 3 trial (*N* = 3297) nor was this observed with oral semaglutide in PIONEER-6 [15] (HR 0.76; 95% CI 0.37–1.56). Similarly, fatal and nonfatal MI were not reduced significantly in SUSTAIN-6 (HR 0.81; 95% CI 0.57–1.16) or PIONEER-6 (HR 1.04; 95% CI 0.66–1.64).

A recent observational study from SWEDEHEART of patients with DM surviving their first MI found that patients taking a GLP-1 RA (86.6% on liraglutide) compared with standard diabetes care had numerically lower rates of myocardial reinfarction (HR 0.71; 95% CI 0.49–1.04) and stroke (HR 0.42; 95% CI 0.18–1.02) [16]. Although the impact on reinfarction and stroke was similar to that of sotagliflozin in SCORED and SOLOIST [5, 6], patients in this observational study were early post MI and at a high risk of recurrent MI and stroke, and the effects did not reach statistical significance [16].

Thus, despite similarity to SCORED (sotagliflozin) in the magnitude of reduction in both fatal and nonfatal MI and stroke with semaglutide in SUSTAIN-6 [14] and GLP-1 RAs (mainly liraglutide) in the SWEDEHEART observational study [16], the results were not statistically significant. Until further comparative studies are available, it is, however, reasonable to assume that the magnitude of benefit for MI and stroke reduction is similar in patients receiving an SGLT1/2 inhibitor or a GLP-1 RA. Of note, the reduction in HHF with GLP-1 RAs [4], although significant, is less than with the SGLT2is [3] and the SGLT1/2i sotagliflozin [5, 6].

Combining both types of agents may yield additional benefits: in a recent observational propensity-matched study from 3 US claims databases of 12,854 patients with type 2 diabetes who added an SGLT2i or sulfonylurea to baseline GLP-1 RA treatment, adding an SGLT2i produced greater cardiovascular benefit (comprising MI, stroke, all-cause mortality, and HHF) [17]. The magnitude of cardiovascular risk reduction echoed the pivotal cardiovascular outcome trials studying SGLT2is versus placebo, in which the baseline use of GLP-1 RAs was minimal. While the proposed mechanisms associated with the reduction in cardiovascular events with SGLT2is and GLP-1 RAs are complementary, further prospective trials will be required to determine whether or not the addition of an SGLT2i to a GLP-1 RA is additive or synergistic.

Evidence supports significant reduction in cardiovascular and kidney outcomes with both SGLT2is and GLP-1 RAs as well as potential benefit from their combination. Although evidence from preclinical studies suggests that SGLT2is reduce infarct size [18], the reduction in incidence of nonfatal and fatal MI in patients with type 2 diabetes on this treatment is modest, approximately 11% [3]. Rather than a direct effect on platelet activation, thrombus formation, or atherosclerotic plaque stability, this benefit is postulated to result from a reduction in preload with a resultant reduction in myocardial wall tension, myocardial oxygen demands, and a consequential reduction in myocardial ischemia [8]. Overall, SGLT2is have not been found to reduce the incidence of nonfatal and fatal stroke [3]. In contrast, GLP-1 RAs demonstrably reduce the incidence of nonfatal and fatal stroke [4], an effect credited to a reduction in thrombosis and an increase in atherosclerotic plaque stability [19, 20].

Therefore, although these medications provide substantial cardiovascular benefits, their protective effect against MI and stroke is modest given the impact of type 2 diabetes as a risk factor for MI as well as ischemic and hemorrhagic stroke. Patients with type 2 diabetes have greater mortality and worse stroke outcomes compared with patients without type 2 diabetes. MI is the primary cause of death in patients with type 2 diabetes. There is a > 20% risk of developing a first MI within the 10 years of developing type 2 diabetes. In patients who experienced MI, the risk of a recurrent one is > 40% [21]. Thus, strategies to prevent stroke and MI in patients with type 2 diabetes are crucial as we face the projected increase in type 2 diabetes over the next decade. The increased risk of stroke associated with prediabetes brings further urgency to stroke prevention in patients at risk of or with type 2 diabetes [22].

With this in mind, we point to the relative increase in SGLT1 vs SGLT2 inhibition with sotagliflozin resulting in a greater reduction in MI and stroke in SCORED [5] compared with the results of the meta-analysis of SGLT2is which failed to show a reduction in stroke [3] and a similar reduction as the GLP-1 RAs [4] but with a greater reduction in HHF. In SCORED [5], sotagliflozin reduced total fatal and nonfatal MI by 32% (HR 0.68, 95% CI 0.52–0.89, *p* = 0.004), total fatal and nonfatal stroke by 34% (HR 0.66, 95% CI 0.48–0.91, *p* = 0.012), and HHF and urgent visits for HF by 33% (HR 0.67; 95% CI 0.55–0.82, *p* ≤ 0.001).

The mechanisms associated with the development of MI and stroke in patients with type 2 diabetes include insulin resistance, increased formation of advanced glycation end products (AGEs), activation of protein kinase C isoforms, over activity of the hexose amine pathway, vascular calcification, an increase in reactive oxygen species (ROS), decreased nitric oxide availability, endothelial dysfunction, inflammatory cytokine activation, increased vascular stiffness, platelet activation, an increased risk of thrombosis, and an increase in plasma and blood viscosity as well as autonomic dysfunction [23, 24]. Chronic kidney disease (CKD) has been recognized as an important risk factor for stroke. However, recent studies suggest that the relationship between CKD and stroke is confounded by its connection to longstanding hypertension [25].

The mechanisms accounting for the reduction in MI and stroke with sotagliflozin in the SCORED [5] and SOLOIST trials [6] remain undetermined. SGLT1 is expressed not only in the brush border of the small intestine and proximal renal tubule but also in salivary glands, liver, pancreatic alpha cells, lungs, heart, skeletal muscle, brain, cervix of the uterus, stomach, mesenteric adipose tissue, and in capillaries of the heart and skeletal muscle [26]. A Mendelian randomization study examining the missense variants in SLC5A1, which is associated with a decrease in SGLT1 function, has shown decreased incidence of type 2 diabetes, obesity, heart failure, and death. Visceral obesity is a risk factor for MI and stroke, and it may cause an increase in inflammatory cytokines, leptin, endothelial dysfunction, and thrombosis [27]. SGLT1 levels are increased in patients with type 2 diabetes [28]. Selective SGLT1 inhibition in the early intestine results in increased glucose delivery to the distal intestine and colon [29–32], where it reduces colonic pH and is metabolized by the gut microbiome, resulting in an increase in short chain fatty acids (SCFA) [33]. A reduction in intestinal glucose absorption and its increased delivery to the distal intestines as a result of SGLT1 inhibition is also linked to a sustained increase in GLP-1 [31]. An increase in native GLP-1 can suppress thrombus growth at both venous and arterial shear rates [19] as well as increase atherosclerotic plaque stability [20]. In view of the reduction in incidence of MI and stroke in SCORED and SOLOIST [5, 6]—greater than that seen with the SGLT2is [3] and many GLP-1 RAs [4, 34]—it is likely that mechanisms other than reduction in blood pressure, visceral obesity, myocardial oxygen demand, and increase in GLP-1 explain this reduction. Other aspects of SGLT1 inhibition may also play an important role.

The development of MI and stroke could be affected by alterations in the intestinal microbiome as a result of SGLT1 inhibition and an increase in delivery of glucose to the distal intestines. Alterations in the gut microbiome associated with obesity and type 2 diabetes trigger inflammation, intestinal permeability, and insulin sensitivity [35]. Aging can cause a decrease in SCFAs in the intestinal microbiome, which is linked to an increase in inflammatory cytokines and weakening of immune defenses [36]. An increase in bacterial lipopolysaccharides (LPS) is associated with an increase in coagulation. LPS binds to toll-like receptors to activate endothelial cells and platelets, leading to activation of the coagulation cascade [37]. The microbiome also produces trimethylamine-N-oxide (TMAO), which causes an increase in platelet activation, thrombosis, and cardiovascular risk [38]. TMAO levels are increased at admission in patients with ischemic stroke and then decrease after 48 h [39]. An increase in TMAO levels is also associated with an increase in 5-year all-cause mortality in patients with stable coronary artery disease [40].

Alterations in the intestinal microbiome as a result of SGLT1 inhibition are of potential importance and deserve further exploration. However, the expression of SGLT1 in the brain and heart [26] is likely connected to the effect that SGLT1 inhibition with sotagliflozin has on stroke and MI.

Cerebral ischemia increases the permeability of the blood–brain barrier (BBB), which increases cerebral edema [41]. SGLT1 expression is increased in cultured endothelial cells from small vessels in the bovine brain under hypoxic conditions [42]. Administration of intracerebral ventricular phlorizin [43, 44] as well as intracerebral ventricular administration of antisense SGLT1mRNA [45] reduced infarct size and cognitive deficits after medial cerebral artery occlusion (MCAO).

Cardiac overexpression of SGLT1 increases myocyte size, collagen 1 gene expression, and interstitial fibrosis in mouse hearts subjected to ischemia, independent of glucose [46]. SGLT1 knockdown demonstrably protected the heart from ischemic-reperfusion injury in mice [47]. Importantly, pretreatment of mice with the selective SGLT1 inhibitor KGA-2727 protected against cardiac remodeling and heart failure in mice after left anterior descending coronary artery occlusion [46]. Phlorizin, a non-selective SGLT inhibitor shown to also block SGLT1, increases infarct size in mice [48]. However, phlorizin’s effect on ischemic-reperfusion injury has been suggested to be due to its effects on glucose transporters in contrast to its effect on SGLT1 [47].

The undetermined mechanisms linking SGLT1 inhibition to MI and stroke necessitate further exploration. Considering the effect of SGLT1 inhibition on the intestinal microbiome as well as the direct effect of inhibition of SGLT1 expression in the heart and brain, there are several ways that SGLT1 inhibition could favorably alter the risk of thrombosis (Fig. 1).

**Fig. 1:**
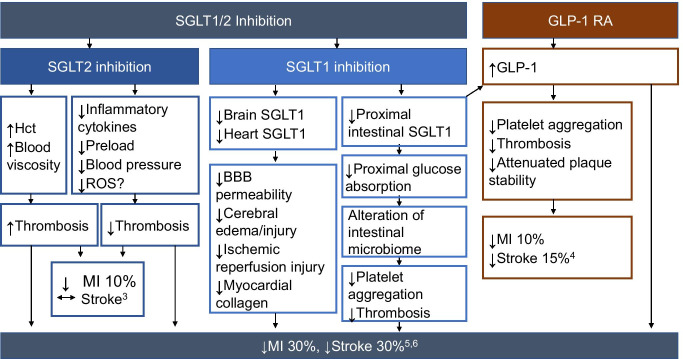
Effects of SGLT1/2 inhibition and GLP-1 receptor agonism on the incidence of myocardial infarction and stroke in patients with type 2 diabetes mellitus. BBB, blood brain barrier; Hct, hematocrit; MI, myocardial infarction; ROS, reactive oxygen species

The adverse effects of adding SGLT1 to SGLT2 inhibition with sotagliflozin resemble those of the SGLT2is alone. All these drugs increase the risk of mycotic genital infections, have an increased but rare incidence of diabetic ketoacidosis, and are associated with a transient hemodynamically mediated decrease but long-term benefit in eGFR [3, 5]. As expected from the expression of SGLT1 in the small intestine, sotagliflozin is also associated with a 2.5–2.8% increase in diarrhea as well as a significant increase in volume depletion (1.3%) and hypotension [5, 6].

Larger and longer studies are required to confirm the effectiveness of combined SGLT1/SGLT2 inhibition in reducing MI and stroke in patients with type 2 diabetes and elucidate the mechanisms associated with this finding. Thus far, the results from SCORED and SOLOIST suggest that an increase in SGLT1 inhibition when added to SGLT2 inhibition may contribute to the reduction in MI and stroke in patients with type 2 diabetes [5, 6]. This benefit is beyond what SGLT2is alone can accomplish and at least similar to GLP-1 RAs but with the added benefit of a reduction in hospitalizations and urgent visits for HF. However, the role of SGLT1/2 inhibition with sotagliflozin as an addition to GLP-1 RA in patients with and without type 2 diabetes at increased risk for stroke and MI remains to be studied. Regardless, finding that a relative increase in SGLT1/SGLT2 inhibition reduces the risk of MI and stroke as well as hospitalizations and urgent visits for heart failure provides an opportunity to further improve quality of life for patients and reduce the healthcare burden associated with type 2 diabetes [49].

## Data Availability

No new data were generated or analyzed in support of this review.
