# Correction to: The Role of Combined SGLT1/SGLT2 Inhibition in Reducing the Incidence of Stroke and Myocardial Infarction in Patients with Type 2 Diabetes Mellitus

**DOI:** 10.1007/s10557-021-07308-6

**Published:** 2021-12-24

**Authors:** Bertram Pitt, Gabriel Steg, Lawrence A. Leiter, Deepak L. Bhatt

**Affiliations:** 1 University of Michigan, Ann Arbor, MI USA; 2 Université de Paris, Hopital Bichat, Paris, France; 3 Li Ka Shing Knowledge Institute, St. Michael’s Hospital, University of Toronto, Toronto, ON Canada; 4 Brigham and Women’s Hospital Heart & Vascular Center and Harvard Medical School, 75 Francis Street, Boston, MA 02115 USA

**Correction to** **:** **Cardiovascular Drugs and Therapy**

https://doi.org/10.1007/s10557-021-07291-y

The original article has been corrected. References 5 and 6 have been switched. Reference 5 “Bhatt DL, Szarek M, Steg PG, et al. Sotagliflozin in patients with diabetes and worsening heart failure. N Eng J Med. 2021;384:117–28” should be “Bhatt DL, Szarek M, Pitt B, et al. Sotagliflozin in patients with diabetes and chronic kidney disease. N Eng J Med. 2021;384:129–39.” Reference 6 “Bhatt DL, Szarek M, Pitt B, et al. Sotagliflozin in patients with diabetes and chronic kidney disease. N Eng J Med. 2021;384:129–39” should be “Bhatt DL, Szarek M, Steg PG, et al. Sotagliflozin in patients with diabetes and worsening heart failure. N Eng J Med. 2021;384:117–28.”

5. Bhatt DL, Szarek M, Pitt B, et al. Sotagliflozin in patients with diabetes and chronic kidney disease. N Eng J Med. 2021;384:129–39.

6. Bhatt DL, Szarek M, Steg PG, et al. Sotagliflozin in patients with diabetes and worsening heart failure. N Eng J Med. 2021;384:117–28.

